# The Relationship between Postexercise Hypotension and Heart Rate Variability before and after Exercise Training

**DOI:** 10.3390/jcdd10020064

**Published:** 2023-02-03

**Authors:** Burak T. Cilhoroz, Amanda Zaleski, Beth Taylor, Antonio B. Fernandez, Lucas P. Santos, Thijs Vonk, Paul D. Thompson, Linda S. Pescatello

**Affiliations:** 1 Department of Exercise Science, Falk College of Sports and Human Dynamics, Syracuse University, Syracuse, NY 13244, USA; 2 Department of Kinesiology, University of Connecticut, Storrs, CT 06269, USA; 3 Department of Preventive Cardiology, Hartford Hospital, Hartford, CT 06106, USA; 4 Exercise Pathophysiology Laboratory, Clinical Research Center, Hospital de Clínicas de Porto Alegre, Porto Alegre 90035-003, Brazil; 5 Department of Physiology, Radboud University Medical Center, 6500 HB Nijmegen, The Netherlands

**Keywords:** autonomic activity, blood pressure, graded exercise stress test, hypertension

## Abstract

Because data are scarce, we examined the relationship between postexercise hypotension (PEH) and heart rate variability (HRV) before and after aerobic exercise training among adults with hypertension. Participants completed a 12 w aerobic training program. Before and after training, they performed a peak graded exercise stress test (GEST) and nonexercise control (CONTROL) and were left attached to an ambulatory BP monitor. Prior to CONTROL, HRV was measured supine for 5 min using a 12-lead electrocardiogram (ECG). The participants (*n* = 18) were middle-aged (52.1 ± 11.7 y) and 50% men with hypertension (131.7 ± 9.8/85.9 ± 8.5 mmHg) and obesity (30.0 ± 3.7 kg·m^−2^). Before training, ambulatory systolic BP (ASBP) and diastolic ABP (ADBP) decreased by 3.2 ± 2.1 mmHg and 2.5 ± 1.5 mmHg, respectively, from baseline after the GEST versus CONTROL (*p* < 0.05). After training, ASBP tended to decrease by 3.5 ± 2.2 mmHg (*p* = 0.055) and ADBP decreased by 1.7 ± 2.5 mmHg (*p* = 0.001) from baseline after the GEST versus CONTROL. Before training, HRV high frequency (HFms^2^) (β = −0.441), age (β = 0.568), and resting SBP (β = 0.504) accounted for 66.8% of the ASBP response (*p* = 0.001), whereas the low frequency (LF)/HF ratio (β = 0.516) and resting DBP (β = 0.277) accounted for 35.7% of the ADBP response (*p* = 0.037). After training, the standard deviation of NN intervals (SDNN) (β = −0.556), age (β = 0.506), and resting SBP (β = 0.259) accounted for 60.7% of the ASBP response (*p* = 0.004), whereas SDNN (β = −0.236) and resting DBP (β = 0.785) accounted for 58.5% of the ADBP response (*p* = 0.001). Our preliminary findings show that adults with hypertension and parasympathetic suppression (i.e., lower SDNN and HFms^2^ and higher LF/HF) may elicit PEH to the greatest degree independent of training status versus adults with parasympathetic predominance, suggesting that resting HRV may be an important determinant of PEH.

## 1. Introduction

Hypertension is the most prevalent cardiovascular disease (CVD) risk factor, affecting ~105 million Americans (~47%) [1,2]. Participation in regular exercise reduces blood pressure (BP) on average by 5–8 mmHg among individuals with hypertension [1,3]. This reduction in BP mitigates the risk of all-cause mortality by 11%, cardiovascular mortality by 16%, coronary heart disease by 18%, and stroke by 36% [4]. Therefore, exercise is recommended as essential lifestyle therapy for the primary prevention, treatment, and control of hypertension [1,3,5,6]. Aerobic exercise immediately reduces BP after a single isolated exercise session [7,8,9,10], termed postexercise hypotension (PEH). PEH persists for 24 h after the exercise bout and is correlated with the BP response to exercise training [11,12,13].

No consensus exists on the precise mechanisms by which PEH occurs. However, it has been hypothesized that most agree that PEH occurs owing to the lasting reductions in total peripheral resistance without adjacent increases in cardiac output among otherwise healthy individuals with hypertension [7,8,14]. Suppression of sympathetic activity deriving from baroreflex resetting after exercise adds to these reductions in total peripheral resistance that is thought to generate PEH response [7,8]. The interaction between sympathetic and parasympathetic activity (i.e., sympathovagal balance), a key factor influencing systemic vascular resistance, can be assessed using heart rate variability (HRV) [15].

Our recent pilot study among firefighters with hypertension showed that the HRV low frequency (LF)/high frequency (HF) ratio, a marker of sympathovagal balance, accounted for ~85% of the BP response after an acute bout of vigorous physical exertion [15]. In addition, Grassler et al. [16] reported that HRV time domain measures were inversely, and frequency domain measures including the LF/HF ratio were directly associated with the BP response following moderate-intensity aerobic exercise training among healthy adults. These authors concluded that exercise training led to decreases in sympathetic activity (as quantified by reduced HRV) along with reductions in BP [16]_._ Enhanced endothelial function and vascular distensibility (i.e., increased ability of large vessels to absorb sympathetic outflow) following exercise training may also account for decreases in sympathetic activity and BP [17]. Hence, the exercise-training-induced suppression of sympathetic activity or enhancement of parasympathetic modulation, or both, may partially mediate the reductions in BP following exercise training.

Surprisingly, the relationship between the time and frequency domain measures of HRV and the BP responses to acute (i.e., PEH) and chronic exercise have not been investigated among the same individuals with hypertension in a within-subject study design. Therefore, we sought to examine the relationship between HRV and PEH before and after aerobic exercise training among adults with hypertension. We hypothesize that adults with hypertension with sympathetic predominance at rest as assessed by HRV will exhibit larger PEH response before and after training.

## 2. Methods

### 2.1. Study Overview

The current study is a substudy of a larger randomized control trial (ClinicalTrials.gov Identifier: NCT03780309) [18]. Participants were recruited from BP screenings, media advertisements, social media, previous studies, and places of work and college campuses with the posting of flyers, class announcements, and newsletters. We prioritized recruiting individuals in the early stages of hypertension as lifestyle interventions such as exercise are critical to prevent the progression of hypertension and its associated outcomes.

**Figure 1:**
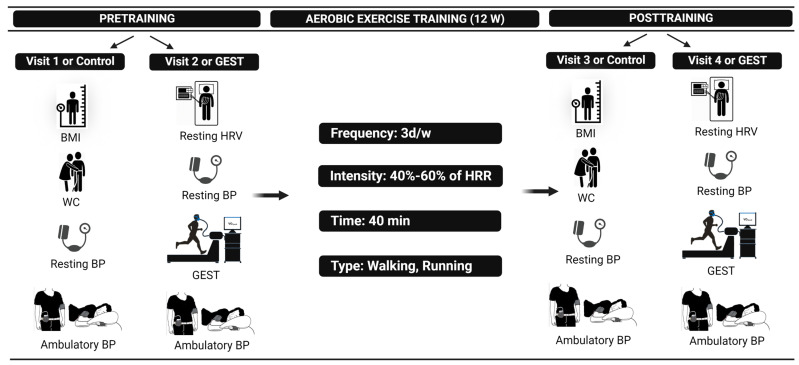
Study design overview. BMI, body mass index; BP, blood pressure; GEST, graded exercise stress test; HRR, heart rate reserve; HRV, heart rate variability; PEH, postexercise hypotension; WC, waist circumference.

The study overview is depicted in Figure 1. Participants were sedentary, physically inactive (i.e., exercising ≤ 2 d/w) (*n* = 18) adults of at least 18 years with elevated BP to established hypertension, defined as having systolic BP (SBP) ≥ 120 to <160 and/or diastolic (DBP) ≥ 80 to <100 mmHg for those not taking medications or SBP < 160 mmHg and DBP < 100 mmHg for those taking medications [2,18] (Table 1). All participants signed an informed consent approved by the University of Connecticut and Hartford Hospital IRBs. The participants attended four laboratory visits with Visits 1 and 2 occurring before training and Visits 3 and 4 after training [18]. All visits were performed in random order at the same time of day separated by at least 48 h to control for any potential influence visit order and circadian variation may have on BP. In Visits 1 and 3 (CONTROL), resting BP, body mass index (BMI), and waist circumference (WC) were measured, while in Visits 2 and 4 (GEST) resting BP, resting HRV, and a peak cardiopulmonary graded exercise stress test (GEST) were performed (Figure 1) [18]. Following all visits, the participants were attached to an ambulatory BP (ABP) monitor for 19 h until the next morning.

### 2.2. Study Procedures

#### 2.2.1. Control Visits 1 and 3

Height and weight were obtained through a calibrated physician balance beam scale to calculate BMI. Then, WC was measured at the height of the iliac crest with a Gulick tape measure. After at least 15 min of seated rest, resting BP was measured, three times 5 min apart according to American Heart Association standards [2] using a BpTRU monitor (BpTRU Medical Devices; Coquitlam, BC, Canada) [18]. Later, an ABP monitor (Oscar2 automatic noninvasive ABP monitor, Suntech Medical Instruments Inc., Raleigh, NC, USA) was attached to the participants’ nondominant arm with a calibration check performed by a mercury sphygmomanometer [18]. The ABP monitor was programmed to acquire three ABP measurements for each waking hour and two for each sleeping hour over 19 h [18]. The participants were asked to continue their normal activities, but not to exercise, and keep their arm straight and parallel to the body while each ABP reading was being taken [18]. They were provided with a journal to note activities performed during each measurement, any unexpected physical or emotional events, and sleep and wake times.

The next morning, the participants returned the ABP monitor along with a journal to the study investigators at Hartford Hospital in their own time. The research assistant inspected the journal and ABP recordings after each visit for any unexpected physical or emotional events that might have influenced recordings. We excluded ABP recordings of SBP > 220 or <80 mmHg or DBP > 130 or <40 mmHg in line with the manufacturer’s exclusion criteria. ABP reports were credible if we obtained at least 80% of the BP recordings [15,18]. The ABP data were averaged over hourly intervals for the awake, sleep, and 19 h [15,18].

#### 2.2.2. Graded Cardiopulmonary Exercise Stress Test Visits 2 and 4

Prior to the GEST, the resting HRV assessments were performed for 5 min in the supine position in a quiet, low-light, and temperature-controlled room after a 5 min rest period using a 12-lead electrocardiogram (ECG) (GE Healthcare, Wauwatosa, WI, USA). The recordings were obtained at a sampling frequency of 1000 Hz. R-R intervals from the ECG were downloaded to a computer in Cardiology XML format. Kubios HRV Premium 3.4.1 (Biomedical Signals Analysis and Medical Imaging Group, University of Kuopio, Finland) was used to analyze and correct the selected segments of ECG R-R intervals to obtain the HRV time and frequency domain measures. We recently showed the threshold-based correction method of the Kubios Premium is a superior artifact correction method compared with the native automatic correction method when analyzing HRV [19]. Therefore, artifacts were edited using the threshold-based correction method of Kubios HRV Premium 3.4.1.

The time domain measures comprised the standard deviation of normal R-R intervals (SDNN), root mean square of successive differences in normal R-R intervals (RMSSD), and the percentage of successive normal R-R intervals greater than 50 ms (pNN50%). The autoregressive model was used to quantify power spectrum density into absolute LF (LF ms^2^: 0.04–0.15 Hz) and HF (HF ms^2^: 0.15–0.40 Hz) domains [15,19]. In addition, normalized LF (LF nu), normalized HF (HF nu), and the LF/HF ratio were calculated. The nonlinear measure of sample entropy (SampEn) was also included in the analysis.

The participants were then prepared to perform the GEST to collect expired oxygen and carbon dioxide via the breath-by-breath collection method (ParvoMedics TrueOne 2400 metabolic cart, Parvomedics Corp., Sandy, UT, USA). After several minutes of seated resting, the GEST was started using the Balke protocol on a Trackmaster treadmill (Full Vision, Inc., Newton, KS, USA) to measure peak oxygen consumption (VO_2_peak) [20]. The Borg rating of perceived exertion (RPE) on the 6 to 20 scale [21] and exercising BP were recorded every 3 min, while HR was measured continuously [18]. Maximum heart rate (HR_max_) was defined as the highest HR recorded by ECG during the GEST. The resting and maximum HR established in the GEST (Visit 2) were used to determine the exercise training intensity workloads [18]. The participants trained at 40–60% of their heart rate reserve (HRR) [18]. Hence, the HR_max_ obtained from Visit 2 was inserted into the HRR formula as follows: HR_rest_ + (HR_max_ − HR_rest_) × 0.40 and 0.60 = HRR. At the conclusion of the GEST visits, the participants were attached to the same ABP monitor following the same protocol as Visits 1 and 3 [18].

#### 2.2.3. Exercise Training

The training has been described in detail in our previous work [18]. Briefly, all participants performed a progressive, supervised, moderate intensity (i.e., 40–60% of HRR) aerobic exercise training for 40–50 min/session, 3 d/w for 12 w [18]. The duration of each exercise session gradually progressed from 15 to 40 min during the first 4 w of training [18]. An additional 5 min warmup and cooldown were added so that the total time of each workout progressed to a total of 50 min [18]. Exercise training was performed at 40%–60% of HRR defined as “moderate” intensity by the American College of Sports Medicine (ACSM) [6] (Figure 1). Walking or jogging on a treadmill was the primary mode of exercise training. The participants also had the option to perform cycling exercises on a Monark 893E Digital Cycle Ergometer (Stockholm, Sweden) to reduce the possibility of overuse injuries and avoid boredom with performing the same exercise modality [18].

The dropout rate of the training program was described as missing >72% of the possible number of 36 exercise sessions (i.e., failing to participate in ≥10 of the total number training sessions) and/or failing to join in 6 successive sessions [18,22]. We reached out to the participants through email and/or phone calls if they started missing weekly supervised exercise sessions of 3 per w for 12 w. We then developed a plan together to have them back on their training schedule and encouraged those who knew in advance that they would miss a few of the exercise sessions to train 4 d/w for several weeks to make up for the missed sessions [18].

### 2.3. Statistical Analysis

Descriptive statistics were calculated on all study variables, and data are reported as mean ± SEM. Shapiro–Wilk tests were used to determine whether data were normally distributed. ABP values were averaged over hourly intervals for the “awake” (hours 1–10), “sleep” (hours 11–19), and “19 h” (hours 1–19). The ABP changes in response to the GEST versus CONTROL were calculated as follows: [(GEST BP at each hourly interval—GEST BP baseline)—(CONTROL BP at each hourly interval—CONTROL BP baseline)] [15]. To assess the ABP response to aerobic exercise training, repeated measures analysis of covariance (RMANCOVA) tested ABP during CONTROL Visits 1 and 3 before versus after exercise training at hourly intervals over 19 h with baseline ABP as a covariate. To assess PEH before and after the training, repeated measures ANCOVA tested pre- versus post-training ABP between the GEST versus CONTROL at hourly intervals over 19 h with baseline ABP as a covariate. Paired-samples t tests tested changes in baseline WC, BMI, VO_2_peak, resting HRV, resting HR, resting BP measured by auscultation in the laboratory, and CONTROL ABP over the awake, sleep, and 19 h before (Visit 1) versus after (Visit 3) training. Multiple variable regression examined predictors of the BP response before and after exercise training (e.g., resting, SDNN, HFms^2^, LF/HF, resting BP, baseline BMI, and age) to study the relationship between the ABP response to the GEST versus CONTROL (i.e., PEH) and resting HRV before and after exercise training. Fixed factors of sex and medication use were also tested in the regression models, but the results remained the same. All statistical analyses were performed with the Statistical Package for Social Sciences Version 20.0 for Windows (SSS, Inc., Chicago, IL, USA) with *p* < 0.05 established as the level of statistical significance.

## 3. Results

All data used in the results are shared in the Supplementary Material. The participants from the larger randomized controlled trial [18] were excluded in the current investigation as they did not complete a baseline measurement of ECG for HRV analysis of which there were six. The remaining participants with an ECG measurement (*n* = 18) were overweight to obese, mostly white, middle-aged men and women with normal HR and stage 1 hypertension. Half of the sample was taking antihypertensive medications that included angiotensin II receptor antagonists, angiotensin-converting-enzyme inhibitors, and diuretics (Table 1). Pre-exercise training VO_2_peak was “fair” according to the ACSM reference standards for men and women of this age [6] (Table 1). The increase in relative VO_2_peak (2.7 ± 6.7 mL/kg/min) was not significant following 12 w of aerobic exercise training (*p* = 0.111). However, the ACSM age-and-sex-specific normative VO_2_peak percentiles increased from “fair” to “good” following training [6]. Additionally, resting HR (4.2 ± 7.8 bpm; *p* = 0.034) decreased following training. The markers of body composition, BMI (0.5 ± 3.0 kg/ms^2^; *p* = 0.466) and WC (−0.1 ± 1.9 cm; *p* = 0.722), were not different before versus after training.

### 3.1. Exercise Training Program

Training sessions were performed in consistent environmental conditions with an average room temperature and humidity of 71.4 ± 2.6 °F and 19.6 ± 14.4% [18]. The participants engaged in 35.6 ± 2.8 min of moderate (78.3 ± 4.9% of HR_max_; 61.2 ± 8.2% HRR; 64.3 ± 7.4% of VO_2_peak; and 12.1 ± 1.1 of RPE) treadmill-based aerobic exercise 2.6 ± 0.4 d/w for 12 w [18]. There were no adverse exercise training-related injuries reported throughout the duration of the study [18].

### 3.2. The Blood Pressure Response after GEST versus CONTROL (i.e., PEH)

#### 3.2.1. Pretraining

**Figure 2:**
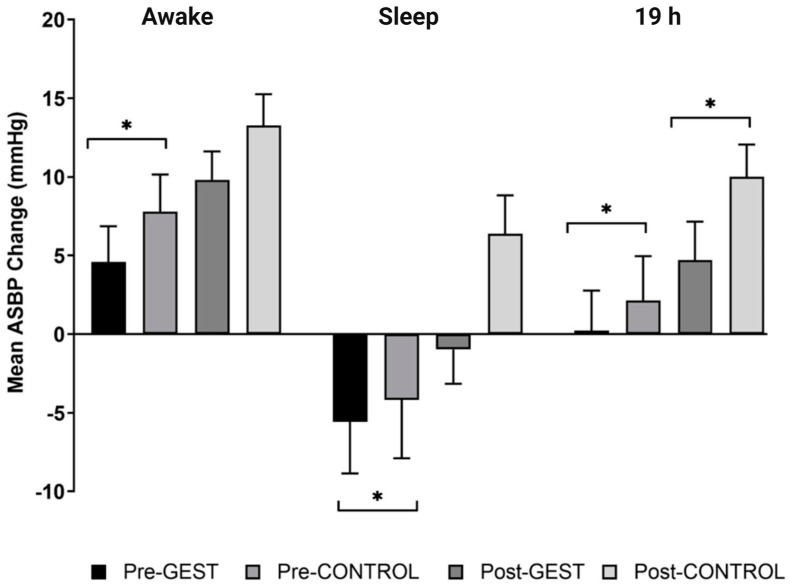
The average ambulatory systolic blood pressure (ASBP) response to the GEST versus CONTROL before and after exercise training over awake, sleep, and 19 h. Pre−GEST: average BP change following the GEST from baseline before the training; Pre−CONTROL: average BP change following CONTROL from baseline before the training; Post−GEST: average BP change following the GEST from baseline after the training; Post−CONTROL: average BP change following CONTROL from baseline after the training; * *p* < 0.05.

**Figure 3:**
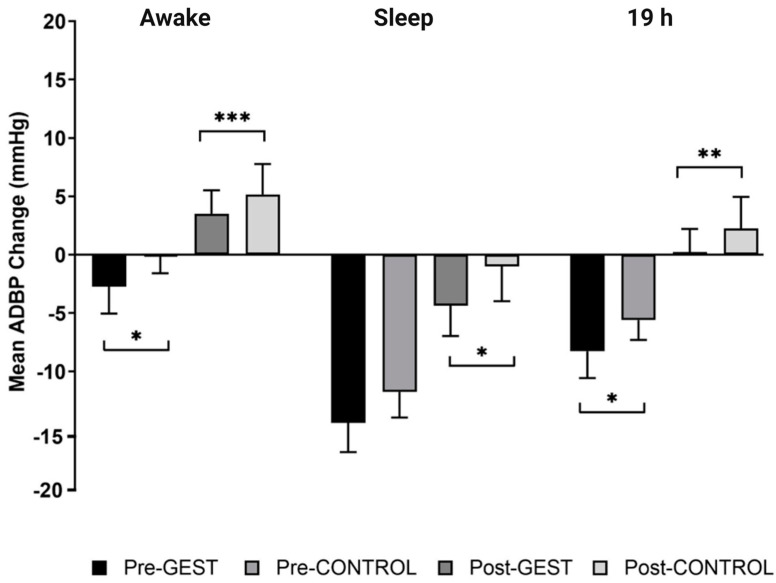
The average ambulatory diastolic blood pressure (ASBP) response to the GEST versus CONTROL before and after exercise training over awake, sleep, and 19 h: Pre−GEST: average BP change following the GEST from baseline before the training; Pre−CONTROL: average BP change following CONTROL from baseline before the training; Post−GEST: average BP change following the GEST from baseline after the training; Post−CONTROL: average BP change following CONTROL from baseline after the training; * *p* < 0.05, ** *p* < 0.01, *** *p* < 0.001 GEST vs. CONTROL.

After versus before the GEST versus CONTROL, ambulatory systolic blood pressure (ASBP) decreased by 3.2 ± 2.1 mmHg (*p* = 0.016) from baseline over awake, 1.4 ± 3.4 mmHg (*p* = 0.043) over sleep, and 2.3 ± 2.4 mmHg (*p* = 0.010) over 19 h (Figure 2). After versus before the GEST versus CONTROL, diastolic ABP (ADBP) decreased by 2.5 ± 1.5 mmHg (*p* = 0.015) from baseline over awake and 2.7 ± 1.5 mmHg (*p* = 0.029) over 19 h (Figure 3). However, ADBP was not different over sleep (−2.9 ± 1.7 mmHg, *p* = 0.206).

#### 3.2.2. Post-Training

After versus before the GEST versus CONTROL, ASBP decreased by 5.3 ± 2.2 mmHg (*p* = 0.028) from baseline over 19 h and tended to decrease by 3.5 ± 2.2 mmHg (*p* = 0.055) over awake and 7.4 ± 3.0 mmHg (*p* = 0.111) over sleep (Figure 2). After versus before the GEST versus CONTROL, ADBP decreased by 1.7 ± 2.5 mmHg (*p* < 0.001) from baseline over awake, 3.4 ± 2.7 mmHg (*p* = 0.015) over sleep, and 2.5 ± 2.4 mmHg (*p* = 0.001) over 19 h (Figure 3).

### 3.3. The Ambulatory Blood Pressure Response to the GEST versus CONTROL (i.e., PEH) after versus before Training

Changes in ASBP awake, sleep, and 19 h following the GEST versus CONTROL after versus before exercise training were not different (*ps* > 0.05) (Table 2). ADBP awake and 19 h was slightly greater following the GEST versus CONTROL after versus before exercise training (*ps* < 0.05), while the decrease in ADBP sleep was not different (*p* = 0.081) (Table 2).

### 3.4. The Ambulatory Blood Pressure Response to Exercise Training

The changes in ASBP awake (pretraining: 142.6 ± 6.8 mmHg, −1.8 ± 1.9 mmHg, *p* = 0.358), sleep (pretraining: 130.3 ± 9.7 mmHg, +3.6 ± 2.8 mmHg, *p* = 0.740), and 19 h (pretraining: 136.8 ± 6.6 mmHg, +0.73 ± 2.2 mmHg, *p* = 0.122) following exercise training were not different. Similarly, the changes in ADBP awake (pretraining: 85.5 ± 4.8 mmHg, −0.18 ± 1.6 mmHg, *p* = 0.912) and 19 h (pretraining: 80.2 ± 4.3 mmHg, +2.2 ± 1.6 mmHg, *p* = 0.184) following exercise training were not different. However, ASBP sleep (pretraining: 74.3 ± 5.5 mmHg, +4.9 ± 1.9 mmHg, *p* = 0.020) increased after exercise training.

### 3.5. The Resting Blood Pressure Response to Exercise Training

Resting SBP and DBP measured in the laboratory by auscultation decreased by 7.9 ± 10.2 mmHg (*p* = 0.004) and 5.6 ± 9.4 mmHg (*p* = 0.023), respectively, after 12 w of exercise training.

### 3.6. The Resting Heart Rate Variability Response to Exercise Training

Aerobic exercise training shifted the sympathovagal balance towards parasympathetic predominance as substantiated by the changes in the HRV time, frequency, and nonlinear measures in the laboratory. Accordingly, the changes in the time domain measures of SDNN (+10.1 ± 1.9 ms, *p* < 0.001), RMSSD (+12.62 ± 3.1 ms, *p* = 0.001), and pNN50 (+8.97 ± 2.7%, *p* = 0.004); the frequency domain measures of absolute LF (−213.5 ± 94.3 ms^2^, *p* = 0.037), HF (+505.0 ± 231.2 ms^2^, *p* = 0.043), and the LF/HF ratio (−2.7 ± 0.5, *p* = <0.001); and the nonlinear measure of SampEn (+0.3 ± 0.3, *p* = 0.003) indicated a shift towards parasympathetic dominance after versus before aerobic exercise training in the laboratory (Table 3).

### 3.7. Relationship between the Ambulatory Blood Pressure Response to the GEST versus CONTROL (i.e., PEH) and Resting Heart Rate Variability before and after Exercise Training

#### 3.7.1. Correlates of the ASBP and ADBP Responses to the GEST versus CONTROL (i.e., PEH) before Training over Awake, Sleep, and 19 h

Before training, as HFms^2^ (β = −0.441) decreased and age (β = 0.568) and resting SBP (β = 0.504) increased, ASBP over awake increased following the GEST versus CONTROL. The regression model combining HFms^2^, age, and resting SBP explained 66.8% of the variance in the ASBP response over the same time period (*p* = 0.001). As the resting SBP (β = 0.596) increased, ASBP over sleep increased following the GEST versus CONTROL. The regression model containing LF/HF (β = −0.178) and the resting SBP and BMI (β = 0.371) accounted for 41.2% of the variance in the ASBP response over the same time period, but this model did not achieve statistical significance (*p* = 0.053). As resting SBP (β = 0.578) and age (β = 0.437) increased, ASBP over 19 h increased following the GEST versus CONTROL. The regression model including SampEn (β = 0.301, *p* = ns) and resting SBP and age accounted for 54.1% of the variation in the ASBP response over the same time period (*p* = 0.010) (Table 4). As LF/HF (β = 0.516) increased, ADBP over awake increased following the GEST versus CONTROL. The regression model comprising LF/HF and resting DBP (β = 0.277, *p* = ns) explained 35.7% of the variance in the ADBP response over awake (*p* = 0.037). As LF/HF (β = 0.473) increased, ADBP over 19 h increased following the GEST versus CONTROL, accounting for 22.3% of the variation in the ADBP response over the same time period (*p* = 0.048). There were no statistically significant models that emerged explaining a clinically important amount of the ADBP response over sleep (Table 5).

#### 3.7.2. Correlates of the ASBP and ADBP Responses to the GEST versus CONTROL (i.e., PEH) after Training over Awake, Sleep, and 19 h

After training, as SDNN (β = −0.556) decreased and age (β = 0.506) increased, ASBP over awake increased following the GEST versus CONTROL. The regression model combining SDNN, age, and resting SBP (β = 0.259, *p* = ns) explained 60.7% of the variance in the ASBP response over the same time period (*p* = 0.004). There were no statistically significant models that emerged explaining a clinically important amount of the ASBP response over sleep and 19 h (Table 4). After training, as the resting DBP (β = 0.785) increased, ADBP over awake increased following the GEST versus CONTROL. The regression model consisting of SDNN (β = −0.236, *p* = ns) and resting DBP explained 58.5% of the variance in the ADBP response over the same time period (*p* = 0.001). As the resting DBP (β = 0.689) increased, ADBP over 19 h increased following the GEST versus CONTROL. The regression model combining LF/HF (β = 0.123, *p* = ns) and resting DBP accounted for 48.2% of the variance in the ADBP response over the same time period (*p* = 0.007). There were no statistically significant models that emerged explaining any meaningful amount of the ADBP response over sleep (Table 5).

### 3.8. Correlates of the Blood Pressure Response to Exercise Training

As the LFnu change (∆LFnu, β = 0.468) decreased, the resting SBP change (∆SBP) decreased after versus before training. The regression model combining the pretraining ASBP awake response to the GEST versus CONTROL (PEH, β = 0.130, *p* = ns), ∆LFnu, and resting SBP (β = 0.415, *p* = ns) explained 43.2% of the ∆SBP (*p* = 0.042) (Table 6). In addition, as the LF/HF change (∆LF/HF, β = 0.716) decreased, the resting DBP change (∆DBP) decreased after versus before training. The regression model comprising the pretraining ADBP awake response to GEST versus CONTROL (PEH, β = 0.287, *p* = ns), LF/HF, and resting DBP (β = 0.264, *p* = ns) accounted for 44.0% of the variation in the ∆DBP (*p* = 0.039) (Table 6).

## 4. Discussion

We examined the relationship between PEH over 19 h and resting autonomic activity as measured by HRV among a small group of adults with hypertension before and after aerobic exercise training. For ASBP, we found that the magnitude of PEH over the awake hours became greater as resting parasympathetic predominance decreased, assessed by lower HFms^2^ and SDNN, before and after training. Before training, resting HFms^2^ age and resting SBP accounted for 67% of the variation in the magnitude of PEH. After training, resting SDNN age and resting SBP explained 61% of the variation in the magnitude of PEH. For ADBP, the magnitude of PEH over the awake hours became greater as resting parasympathetic predominance decreased and/or sympathetic predominance increased, assessed by higher LF/HF, before training only. Before training, resting LF/HF and resting DBP accounted for 36% of the variation in PEH. Similarly, the magnitude of PEH over sleep and 19 h increased as resting parasympathetic predominance decreased before and after training.

In addition to statistical significance, the magnitude of the PEH response observed in our study was also clinically meaningful as reductions in ASBP and ADBP of ≥2 mm reduce the incidence of CVD in both individuals with hypertension and normal BP [23,24]. Similarly, the magnitude of increase in HRV following exercise training has clinical significance as a meta-analytic review by Fang at al. [25] showed that patients with lower HRV have 121% and 46% higher risk of all-cause death and cardiovascular events compared with those with higher HRV. However, the scaling and cutoff values of “low” and “high” were not classified due to the significant heterogeneity that exists across the reported studies. Of note, no subject exhibited a hypertensive response to the GEST despite all having hypertension. The peak SBP and DBP responses of all the subjects to the GEST were < 210 mmHg and <110 mmHg, respectively (see Supplementary Material). Further, the peak SBP and DBP values to the GEST returned to baseline values within an hour following the GEST (see Supplementary Material).

Hypertension is considered the most important global risk factor for CVD morbidity and mortality [26]. Hence, determining the predictors of PEH (i.e., the immediate, prolonged BP reductions that follow a bout of acute exercise) is of clinical importance among adults with hypertension [8,9,10,11,13,27,28,29,30]. Consistent with our hypothesis and our previous work among firefighters with hypertension [15], we found that the magnitude of PEH was greatest among adults with hypertension and reduced parasympathetic predominance at rest independent of training status, which should be confirmed in future larger studies with different volumes and intensities of training. Although assessing vascular function was beyond the scope of the current study, it is important to note that alterations in vascular tone (e.g., increased arterial distensibility) may have also contributed to PEH mediated by ANS as alterations in ANS may be secondary to changes in the vascular system [17]. In addition, resting BP and age were positively associated with PEH before and after training among adults with hypertension. This result with resting BP reinforces reports that those with higher BP benefit the most with the antihypertensive benefits of PEH [4,31,32]. PEH is reported in younger [28,30] and older adults [29,33]. However, our findings suggest that PEH may be more pronounced as adults with hypertension age.

PEH may occur due to reduced total peripheral resistance without a concomitant increase in cardiac output in otherwise healthy individuals with hypertension [8]. The suppression of sympathetic activity resulting from baroreflex resetting following exercise may be a key contributor to the decrease in total peripheral resistance that occurs following exercise [7,8,34]. An intriguing study by Jones and colleagues [35] investigating the age-related differences in autonomic nervous system support (i.e., cardiac vagal inhibition of HR) of BP reinforces our finding of the negative relationship between resting parasympathetic predominance and the magnitude of PEH. Jones and colleagues [35] examined the HR and BP in responses to ganglionic blockade with intravenous trimethaphan in young and older healthy men with elevated BP [8]. The decreases in mean arterial BP to ganglionic blockage were almost twice as great in the older men due to negligible increases in HR and cardiac output [35]. In all subjects, the HR response was positively related to baseline HRV and the HF power of HRV (i.e., HR increased as a function of the increase in baseline HRV), and the reductions in mean arterial BP became greater as baseline BP and sympathetic activity increased and baseline HRV decreased [35]. Therefore, reduced resting parasympathetic predominance (and/or increased sympathetic predominance) and higher BP at rest may have contributed to the greater magnitude of PEH we observed as did increasing age.

Another novel finding is the magnitude of PEH was not different before and after training under ambulatory conditions in the same participants over 19 h. Thus, PEH does not appear to depend on training status, suggesting that sedentary and trained individuals may benefit from its antihypertensive effects. Surprisingly, in contrast to reports in the literature [12,13], we found that manifesting PEH pretraining was not associated with the reductions in resting SBP and DBP that occurred after training. Several studies have reported PEH to be positively correlated with the BP reductions following aerobic exercise training [11,12,13]. Kleinnibbelink et al. [12] investigated young men with normal BP and measured PEH before and after vigorous intensity aerobic exercise training for 60 min in the laboratory. Liu et al. [13] studied middle-aged men and women with elevated BP before and after moderate intensity aerobic exercise training, and measured PEH before and after training for 30 min in the laboratory. Hecksteden et al. [11] studied healthy middle-aged adults with stage 2 hypertension [2] before and after vigorous intensity aerobic exercise training and measured PEH before and after training for 10 min in the laboratory. Together, these research teams studied PEH in the laboratory and did not integrate a control sham session in their study designs to account for the placebo effects of the laboratory on BP or the circadian variation in BP that occurs under ambulatory conditions [36,37]. Reasons for the discrepancy between our findings and those of Kleinnibbelink et al. [12], Liu et al. [13], and Hecksteden et al. [11] are not clear but may be attributed to differences in our experimental designs including the BP status, age, gender of the participants, the type of exercise intervention, and the definition of PEH that was used.

The present study is subject to limitations. Additional factors associated with PEH, such as the properties of the vascular system (e.g., arterial stiffness and compliance), may have contributed to PEH response due to their relationships with ANS. In addition, the small sample size and the lack of sex and racial/ethnic diversity limit the extrapolation of our findings to the general population of adults with hypertension. Further, our correlational findings should be interpreted with caution as they do not imply causality. Nevertheless, the current study is the first rigorously designed substudy of a larger randomized control trial [18] to measure PEH before and after training in the same individuals with hypertension under ambulatory conditions. Our new preliminary findings are, among adults with hypertension, the magnitude of PEH (1) became greater as resting parasympathetic predominance decreased independent of training status, and (2) was not different before and after training under ambulatory conditions in the same participants over 19 h, suggesting that PEH occurs in sedentary and trained adults. Our preliminary findings should be confirmed using different volumes and intensities of training among a larger, more heterogenous sample of adults with hypertension across the life span.

In accordance with our hypothesis, we found that with hypertension, the magnitude of PEH became greater as resting parasympathetic predominance decreased and resting BP increased among a small group of middle-aged adults regardless of training status obtained following 12 w (40–50 min/session, 3 d/w) of moderate-intensity (i.e., 40–60% of HRR) aerobic exercise training. Furthermore, PEH was of similar magnitude before and after training under ambulatory conditions in the same participants over 19 h. Further studies are needed to more definitively determine the characteristics of patients that can maximally benefit from the antihypertensive effects of PEH.

## Figures and Tables

**Table 1:** Baseline participant characteristics (*n* = 18, mean ± SEM).

Variables	Values
Age (year)	51.9 ± 2.8
Sex (% men)	50%
Race (Caucasian/African American/Asian)	15/1/2
Waist circumference (cm)	102.9 ± 2.8
Body mass index (kg/m^2^)	29.4 ± 0.9
Systolic blood pressure (mmHg)	135.3 ± 2.7
Diastolic blood pressure (mmHg)	85.8 ± 1.7
Heart Rate (bpm)	76.3 ± 2.5
Duration of hypertension (year)	6.0 ± 1.3
Medication use (%)	50%
VO_2_peak (mL/kg·min^−1^)	27.3 ± 1.6

**Table 2:** Comparison of the ABP response to the GEST versus CONTROL after versus before training over awake, sleep, and 19 h (Mean ± SEM).

Time	Pretraining *n* = (18)	Post-Training *n* = (18)	Post-Pretraining *n* = (18)	*p*
Awake	−3.2 ± 2.1	−3.5 ± 2.2	−0.3 ± 2.9	0.874
Sleep	−1.4 ± 3.4	−7.4 ± 3.0	−6.0 ± 3.7	0.567
19 h	−2.3 ± 2.4	−5.3 ± 2.2	−3.0 ± 2.8	0.630
Awake	−2.5 ± 1.5	−1.7 ± 2.5	0.9 ± 3.0	0.002
Sleep	−2.9 ± 1.7	−3.4 ± 2.7	−0.5 ± 3.2	0.081
19 h	−2.7 ± 1.5	−2.5 ± 2.4	0.2 ± 2.9	0.010

GEST: graded exercise stress test; CONTROL: nonexercise control; ASBP: ambulatory systolic blood pressure; ADBP: ambulatory diastolic blood pressure; Awake: ambulatory blood pressure values averaged over hourly intervals for hours 1–10; Sleep: ambulatory blood pressure values averaged over hourly intervals for hours 11–19; 19 h: ambulatory blood pressure values averaged over hourly intervals for hours 1–19.

**Table 3:** Resting HRV before and after 12 w of exercise training (*n* = 18, Mean ± SEM).

Variable	Pretraining *n* = (18)	Post-Training *n* = (18)	Post-Pretraining *n* = (18)	*p*
SDNN (ms)	38.2 ± 2.7	48.4 ± 3.0	10.1 ± 1.9	<0.001
RMSSD (ms)	24.0 ± 4.2	36.6 ± 5.3	12.62 ± 3.1	0.001
pNN50%	4.9 ± 2.4	13.9 ± 4.6	8.97 ± 2.7	0.004
LF (ms^2^)	741.2 ± 113.8	527.7 ± 113.0	−213.5 ± 94.3	0.037
HF (ms^2^)	384.0 ± 105.2	889.0 ± 323.6	505.0 ± 231.2	0.043
LF (nu)	65.4 ± 5.0	61.7 ± 5.3	−3.7 ± 4.8	0.452
HF (nu)	34.6 ± 5.0	38.2 ± 5.3	3.7 ± 4.8	0.455
LF/HF	4.0 ± 0.6	1.3 ± 0.2	−2.7 ± 0.5	<0.001
SampEn (ms)	1.1 ± 0.1	1.4 ± 0.1	0.3 ± 0.3	0.003

SDNN: Standard deviation of normal-to-normal RR intervals; RMSSD: root mean square of successive RR interval differences; pNN50%: relative number of successive RR interval pairs that differ more than 50 msec; LFms^2^: low frequency in absolute power; HFms^2^: high frequency in absolute power; LFnu: Low frequency in normalized units; HFnu: high frequency in normalized units; SampEn: sample entropy.

**Table 4:** Correlates of the ASBP response to the GEST versus CONTROL (PEH) before and after training over awake, sleep, and 19 h.

	Pretraining						Post-Raining					
Variable	Predictors	β	t	PVE	*p*	VIF	Predictors	β	t	PVE	*p*	VIF
ASBP (mmHg) Awake				0.668	0.001					0.607	0.004	
	HFms^2^	−0.411	−2.573	0.321	0.022	1.076	SDNN	−0.556	−3.233	0.427	0.006	1.055
	Age	0.568	3.604	0.481	0.003	1.047	Age	0.506	2.768	0.354	0.015	1.188
	SBP	0.504	3.166	0.417	0.007	1.067	SBP	0.259	1.451	0.131	0.169	1.136
ASBP (mmHg) Sleep				0.412	0.053					0.153	0.289	
	LF/HF	0.178	0.846	0.049	0.412	1.060	pNN50	0.197	0.827	0.044	0.421	1.005
	SBP	0.596	2.795	0.358	0.014	1.083	SBP	0.351	1.473	0.126	0.161	1.005
	BMI	0.371	1.792	0.187	0.095	1.022						
ASBP (mmHg) 19 h				0.541	0.010					0.408	0.055	
	SampEn	0.301	1.568	0.139	0.139	1.123	SDNN	0.369	1.714	0.173	0.109	1.055
	SBP	0.578	3.101	0.407	0.008	1.059	Age	0.391	1.747	0.179	0.103	1.188
	Age	0.437	2.307	0.275	0.037	1.095	SBP	0.313	1.427	0.127	0.176	1.136

GEST: graded exercise stress test; CONTROL: nonexercise control; SBP: resting systolic blood pressure taken in the laboratory; ASBP: ambulatory systolic blood pressure; ADBP: ambulatory diastolic blood pressure; BMI: body mass index (kg/m^2^); PVE = portion of variance explained; VIF = variance inflation factor; SDNN: standard deviation of normal-to-normal RR intervals; pNN50%: relative number of successive RR interval pairs that differ by more than 50 msec; LFms^2^: low frequency in absolute power; HFms^2^: high frequency in absolute power; SampEn: sample entropy.

**Table 5:** Correlates of the ADBP response to the GEST versus CONTROL (PEH) before and after training over awake, sleep, and 19 h.

	Pretraining						Post-Training					
Variable	Predictors	β	t	PVE	*p*	VIF	Predictors	β	t	PVE	*p*	VIF
ADBP (mmHg) Awake				0.357	0.037					0.585	0.001	
	LF/HF	0.516	2.487	0.292	0.025	1.002	SDNN	−0.236	−1.382	0.113	0.187	1.059
	DBP	0.277	1.337	0.106	0.201	1.002	DBP	0.785	4.588	0.584	<0.001	1.059
ADBP (mmHg) Sleep				0.136	0.132					0.119	0.388	
	LFnu	0.368	1.585	0.136	0.132	1.000	pNN50	0.377	1.959	0.204	0.069	1.018
							DBP	0.612	3.184	0.403	0.006	1.018
ADBP (mmHg) 19 h				0.223	0.048					0.482	0.007	
	LF/HF	0.473	2.146	0.223	0.048	1.000	LF/HF	0.123	0.663	0.028	0.518	1.002
							DBP	0.689	3.701	0.477	0.002	1.002

GEST: graded exercise stress test; CONTROL: nonexercise control; SBP: resting systolic blood pressure taken in the laboratory; ASBP: ambulatory systolic blood pressure; ADBP: ambulatory diastolic blood pressure; BMI: body mass index (kg/m^2^); PVE = portion of variance explained; VIF= variance inflation factor; SDNN: standard deviation of normal-to-normal RR intervals; pNN50%: relative number of successive RR interval pairs that differ by more than 50 msec; LFnu: low frequency in normalized units; LF/HF: the ratio’s low frequency in absolute power over high frequency in absolute power.

**Table 6:** Correlates of the blood pressure response to exercise training.

Variable	Predictors	β	t	PVE	*p*	VIF
∆SBP (mmHg)				0.432	0.042	
	Pretraining ASBP awake	0.130	0.546	0.021	0.594	1.400
	∆LF nu	0.468	2.175	0.253	0.047	1.114
	SBP	0.415	1.747	0.179	0.103	1.393
∆DBP (mmHg)				0.440	0.039	
	Pretraining ADBP awake	0.287	1.204	0.094	0.249	1.418
	∆LF/HF	0.716	3.154	0.415	0.007	1.289
	DBP	0.264	1.247	0.100	0.233	1.122

SBP: Resting systolic blood pressure taken in the laboratory; DBP: resting diastolic blood pressure taken in the laboratory; ∆SBP: resting systolic blood pressure taken in the lab after versus before training; ∆DBP: resting diastolic blood pressure taken in the lab after versus before training; ∆LFnu: resting low frequency in normalized unit after versus before training; ∆LF/HF: resting low frequency in absolute power over high frequency in absolute power ratio after versus before training.

## Data Availability

The data presented in this study are available in the Supplementary Material.

## Supplementary Materials

The supporting information can be downloaded at: https://www.mdpi.com/article/10.3390/jcdd10020064/s1.
